# Supplementary figures and images for: A Truncated Form of HpARI Stabilizes IL-33, Amplifying Responses to the Cytokine
Source: Front Immunol. 2020 Jun 30;11:1363. doi: 10.3389/fimmu.2020.01363 (PMC7338556; doi:10.3389/fimmu.2020.01363)

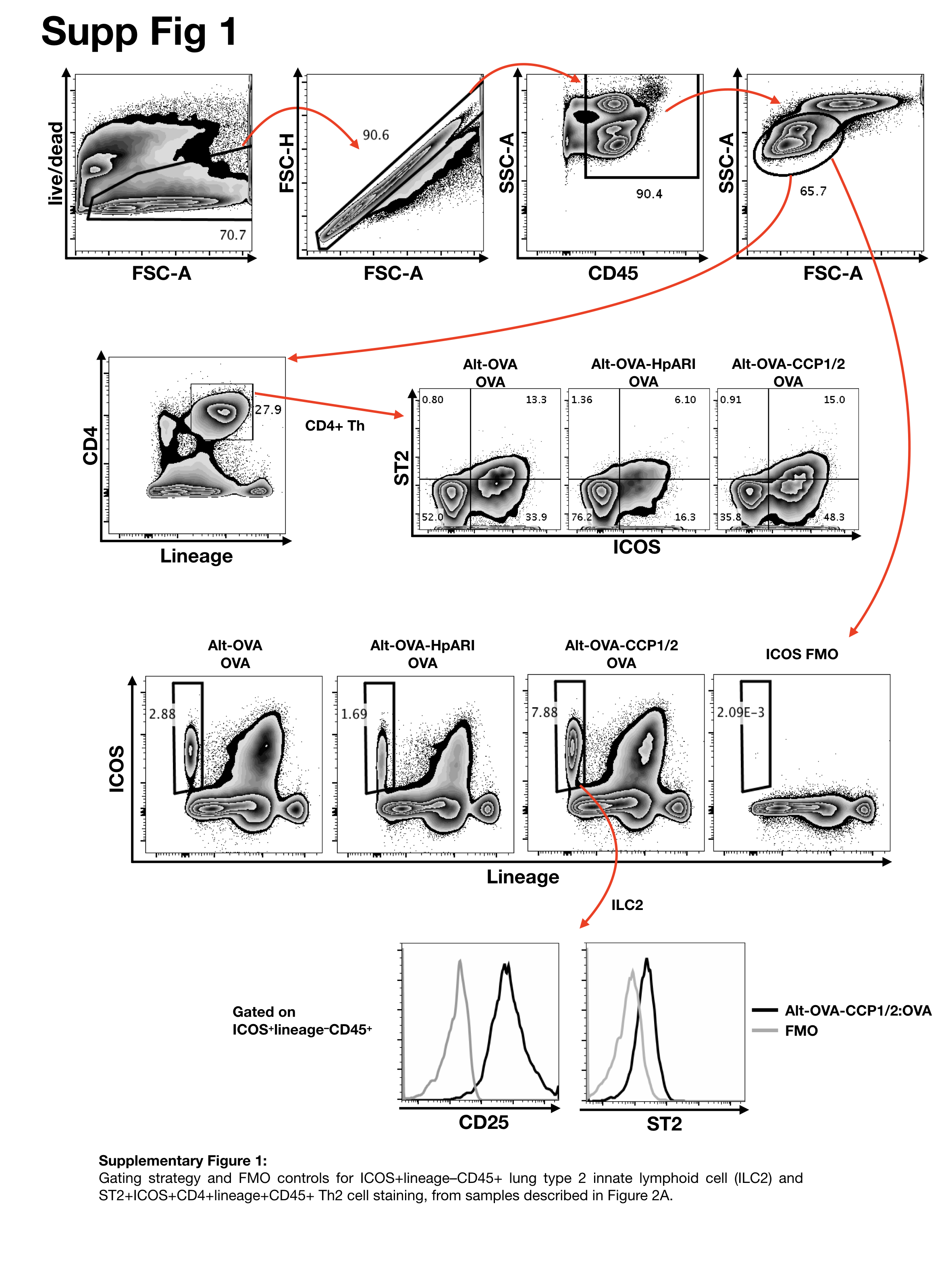

Supplement: Supplementary file 1 [file Image_1.TIFF]

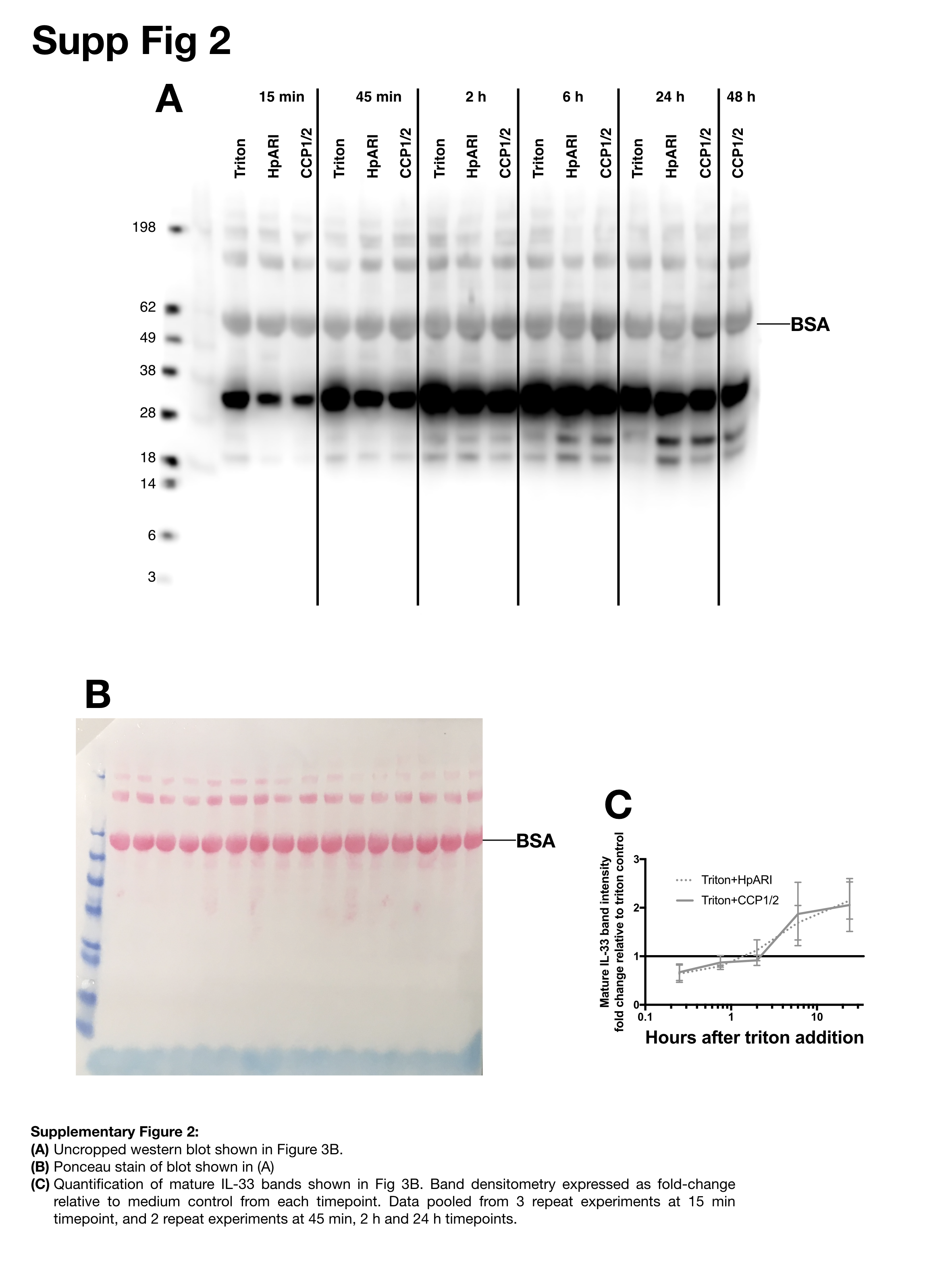

Supplement: Supplementary file 2 [file Image_2.TIFF]

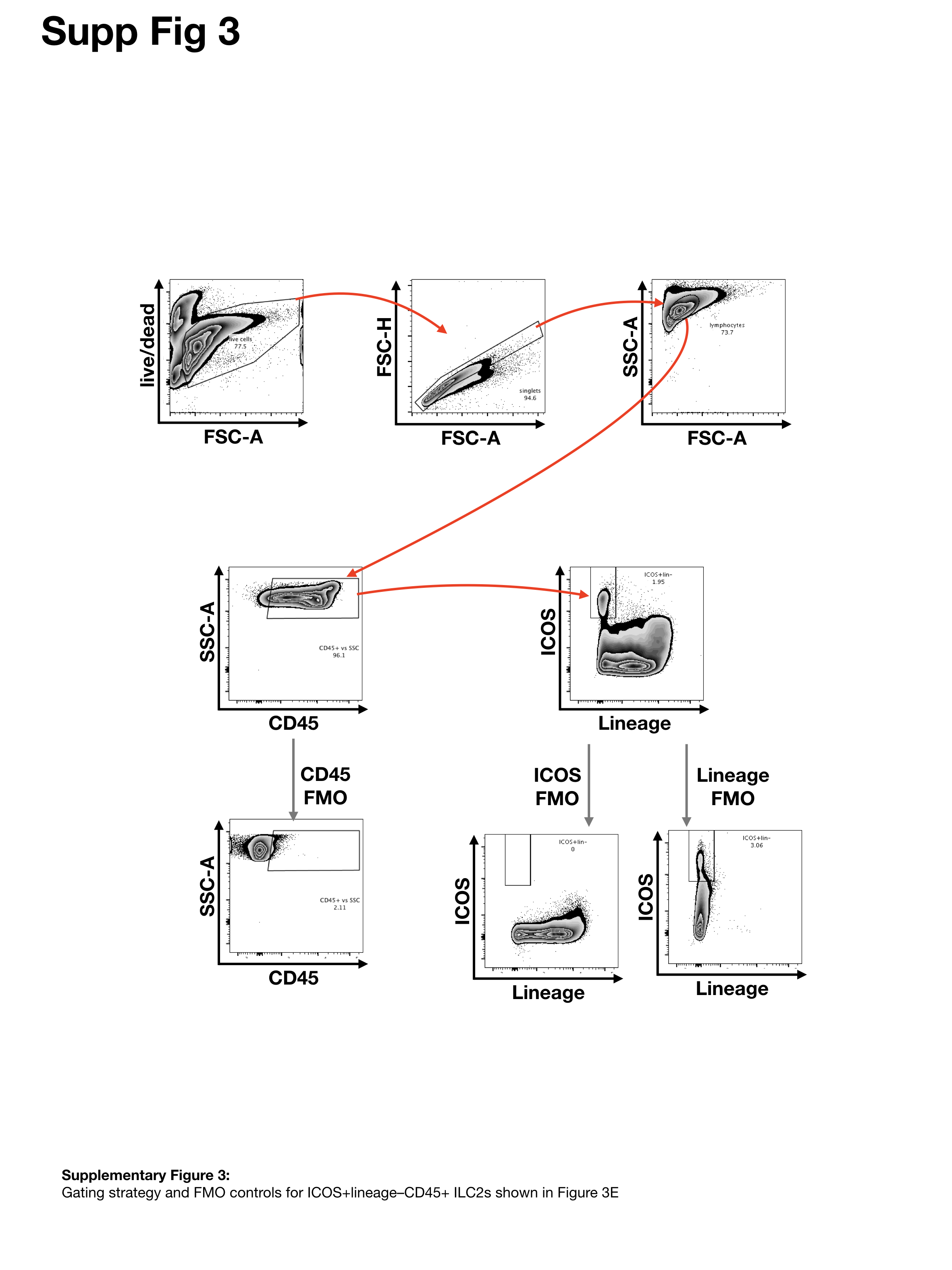

Supplement: Supplementary file 3 [file Image_3.TIFF]
